# Exploring salinity induced adaptations in marine diatoms using advanced photonic techniques

**DOI:** 10.1038/s41598-024-83640-9

**Published:** 2024-12-30

**Authors:** Julijana Cvjetinovic, Yekaterina D. Bedoshvili, Nickolai A. Davidovich, Eugene G. Maksimov, Ekaterina S. Prikhozhdenko, Daria A. Todorenko, Daria V. Bodunova, Olga I. Davidovich, Igor S. Sergeev, Dmitry A. Gorin

**Affiliations:** 1 Center for Photonic Science and Engineering, Skolkovo Institute of Science and Technology, 30 Bolshoy Boulevard, bld. 1, Moscow, 121205 Russia; 2 Limnological Institute, Siberian Branch, Russian Academy of Sciences, 3 Ulan-Batorskaya str, Irkutsk, 664033 Russia; 3 T. I. Vyazemsky Karadag Scientific Station, Natural Reserve of the Russian Academy of Sciences, Kurortnoe, 298188 Feodosiya Russia; 4 Department of Biology, M.V. Lomonosov Moscow State University, 1 Leninskie Gory, Moscow, 119234 Russia; 5 Saratov State University, 83 Astrakhanskaya str, Saratov, 410012 Russia

**Keywords:** Fluorescence lifetime imaging, Photoacoustic imaging, Ultrastructure, Salinity, Diatoms, Optics and photonics, Biophotonics, Biological fluorescence, Cellular imaging

## Abstract

Photonic-based methods are crucial in biology and medicine due to their non-invasive nature, allowing remote measurements without affecting biological specimens. The study of diatoms using advanced photonic methods remains a relatively underexplored area, presenting significant opportunities for pioneering discoveries. This research provides a comprehensive analysis of marine diatoms, specifically *Nitzschia* sp., across varying salinity levels, integrating fluorescence lifetime imaging microscopy (FLIM), combined photoacoustic and fluorescence tomographies (PAFT), and ultrastructural examinations using transmission electron microscopy. Key findings include a systematic shift in the mean fluorescence lifetime from 570 ps at 20‰ to 940 ps at 80‰, indicating functional adaptations in chlorophyll molecules within light-harvesting complexes. At 60‰ salinity, anomalies are observed in the development of silica valves and polysaccharide layers, suggesting abnormalities in valve morphogenesis. Lipid droplets within the cells display a minimum diameter at 40‰, indicating metabolic adjustments to osmotic stress. The intensity of both fluorescence and photoacoustic signals increases with increasing salinity levels. These insights enhance understanding of the ecological implications of salinity stress on diatom communities and pave the way for future research on leveraging the unique adaptive mechanisms of microalgae for environmental monitoring and sustainable biotechnological applications.

**Supplementary Information:**

The online version contains supplementary material available at 10.1038/s41598-024-83640-9.

## Introduction

Diatom algae, crucial components of aquatic ecosystems, play a significant ecological role as primary producers and as a source of approximately 20% of the Earth’s oxygen^1,2^. As primary producers, these single-celled microalgae not only form the backbone of marine food webs but also serve as intricate biofactories. Diatoms from the kingdom Chromista^3^ exemplify nature’s penchant for diversity and precision, as they assimilate silicon, primarily silicic acid, to craft species-specific frustules—silica shells renowned for their ordered complexity and nanoscale intricacy^4^. These nanostructured microobjects have not escaped the attention of the scientific community, finding applications that span from biotechnology to the high-precision realms of microelectronics and medicine, presenting promising templates for advanced material synthesis^5–11^. They also represent renewable resources with potential applications in biofuel production^12^, dietary supplements^13^, animal feed^14^, and as phase materials for creating lightweight and durable composites^15^.

The ability of diatoms to thrive under varying salinity levels is pivotal, as salinity serves as a critical environmental factor influencing their morphological variability, physiological processes, and ultimately, their survival^16^. Extant research delineates that environmental determinants, including salinity fluctuations, impose substantial effects on frustule morphology and nanostructure across various diatom taxa^17,18^. Vrieling et al.^19^ demonstrated that reducing salinity from 28‰ to 20 and 15‰ led to a notable increase in the biogenic silica content per cell in *Thalassiosira weissflogii* and *Navicula salinarum*, which corresponds to a rise in the density of chemically derived silica under conditions of low salinity. Concurrently, at higher salinity levels, there is a modification in the arrangement of silica particles within the developing valves^19^. Moreover, salinity parameters have been observed to influence diatom division rates and the morphological features of their silica frustules^20–22^. The research by Kamakura et al. on *Pleurosira laevis* revealed that even minor fluctuations in salinity trigger substantial transcriptomic responses, uncovering the genetic mechanisms and complex network of cellular processes governing diatom morphology that facilitate morphological adaptation and osmoregulation^22^. Another study showed that certain diatom genera inhabit hypersaline environments up to 205‰, while theoretical salinity limit was 324‰.^23^

Despite this knowledge, existing research has primarily relied on traditional microscopy and chemical analysis, lacking a comprehensive view of these adaptations. Techniques such as laser-based microscopy have pushed beyond traditional limitations, allowing scientists to visualize cellular organelles with unprecedented clarity and transform microscopy from qualitative to quantitative analysis^23^. This includes the use of laser scanning microscopy (LSM)^24^, fluorescence lifetime imaging (FLIM)^25,26^, photoacoustic (optoacoustic) imaging^27^, super-resolution microscopy^28^, and their combinations^29,30^, to achieve molecular-specific imaging. Despite the wide applications of these advanced photonic tools, their use in algae research, particularly in studying diatom algae, remains underexplored. Therefore, this study aims to address this gap by employing non-invasive modern photonic methods alongside ultrastructural and photosynthetic efficiency analyses to explore salinity-induced adaptations in *Nitzschia* sp. at both cellular and subcellular levels. By integrating physiological, structural, and functional insights, we aim to provide a comprehensive understanding of how diatoms adapt to changing salinity ranges from 10 to 150‰, ultimately shedding light on their ecological resilience and biotechnological potential.

LSM serves as a crucial tool for visualization of lipid droplets and silicon assimilation from the medium after diatom staining. Lipid accumulation is an important indicator of metabolic adjustments under stress^31^, while silicon uptake reflects active growth and frustule formation. However, LSM has limitations in quantitatively assessing photosynthetic efficiency or capturing detailed ultrastructural changes, necessitating the use of complementary methods to achieve a comprehensive understanding of cellular adaptations and physiological responses. Through FLIM we observe the effects of salinity on photoprotective mechanisms and energy transfer within photosystem II (PSII). FLIM provides detailed information on fluorescence lifetimes, which are indicative of the conditions within chloroplasts^32^. However, FLIM does not provide spatial resolution of diatom ultrastructure. Combining FLIM with fast chlorophyll *a* fluorescence (OJIP) measurements could offer a more comprehensive approach. OJIP transients provide a dynamic view of the photosynthetic apparatus’s response to environmental factors, such as salinity, offering valuable insights into PSII efficiency^33^.

Photoacoustic imaging can be used to study diatom algae grown under different salinity levels by detecting the optical absorption properties of diatom chromophores^32^. In diatoms, the primary chromophores are chlorophylls and carotenoids, which absorb light and generate photoacoustic signals when exposed to pulsed laser light^32,34^. The intensity of the photoacoustic signal depends on the chromophore concentration, which is influenced by the physiological state of the algae. Photoacoustic and fluorescence tomography (PAFT) non-invasively captures signals from diatoms, providing insights into chromophore distribution, physiological state, and photosynthetic adaptation to salinity changes. The limitation of PAFT lies in its moderate resolution (up to 150 μm), which may not be sufficient for resolving finer ultrastructural details within small diatom cells. Therefore, we also employed transmission electron microscopy (TEM) to achieve higher resolution and conduct ultrastructural examinations of diatom adaptations under different salinity conditions, providing insights into the precise morphological changes that other imaging methods cannot capture^35^.

Together, these imaging methods offer a comprehensive view of diatom physiology by providing insights into photosynthetic efficiency and ultrastructural changes in response to varying salinity. This combined approach allows for an in-depth understanding of diatom adaptations, contributing to knowledge about their resilience and biotechnological applications, such as environmental biosensing and the development of resource-efficient technologies.

**Fig. 1:**
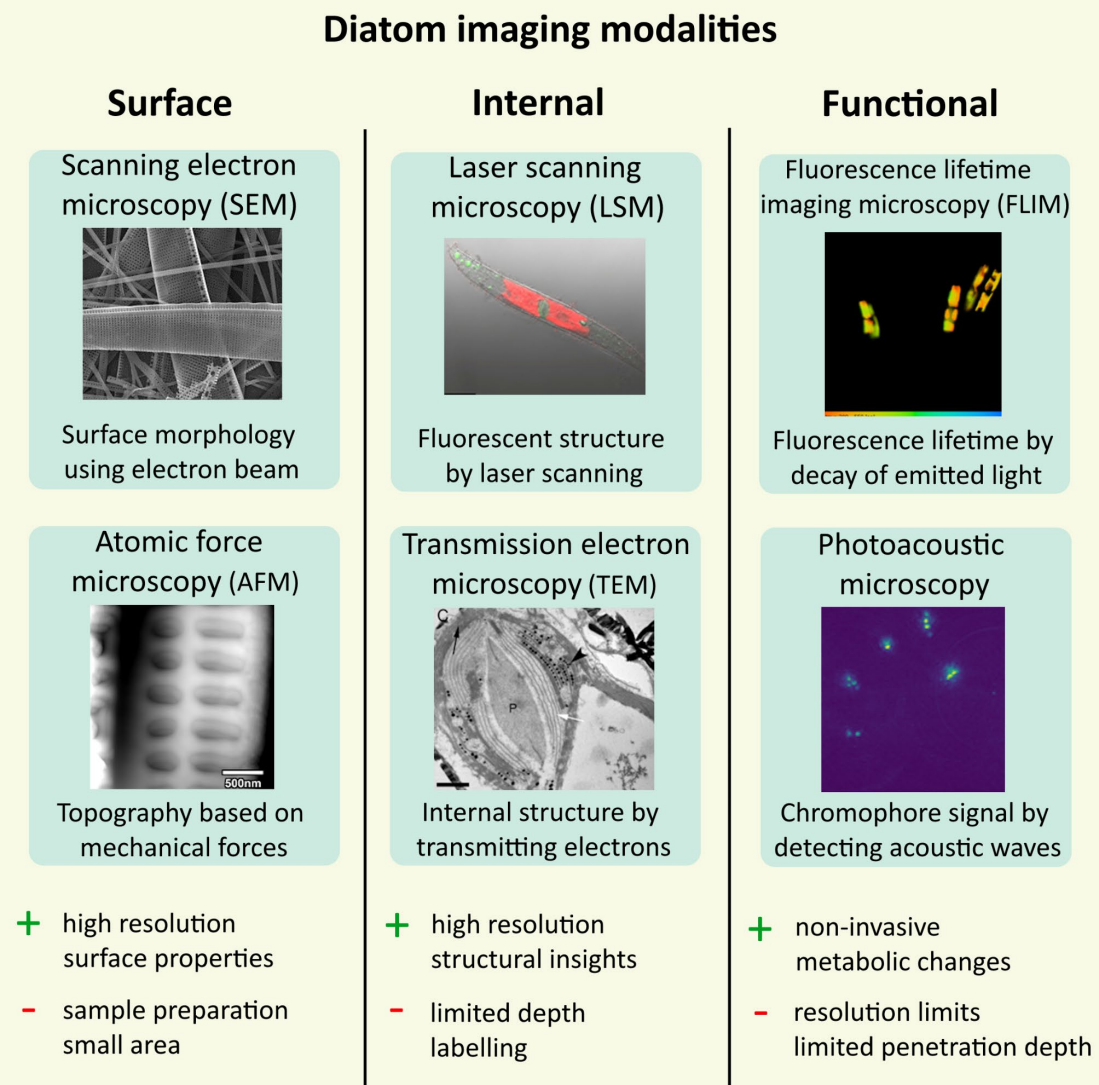
Overview of diatom imaging techniques, categorized by the type of analysis they offer: surface, internal, and functional analysis. Advantages (+) and disadvantages (-) of each imaging modality are listed.

Figure 1 provides an overview of the diverse advanced imaging methods employed to study diatom algae, highlighting their key features and limitations.

## Results

**Fig. 2:**
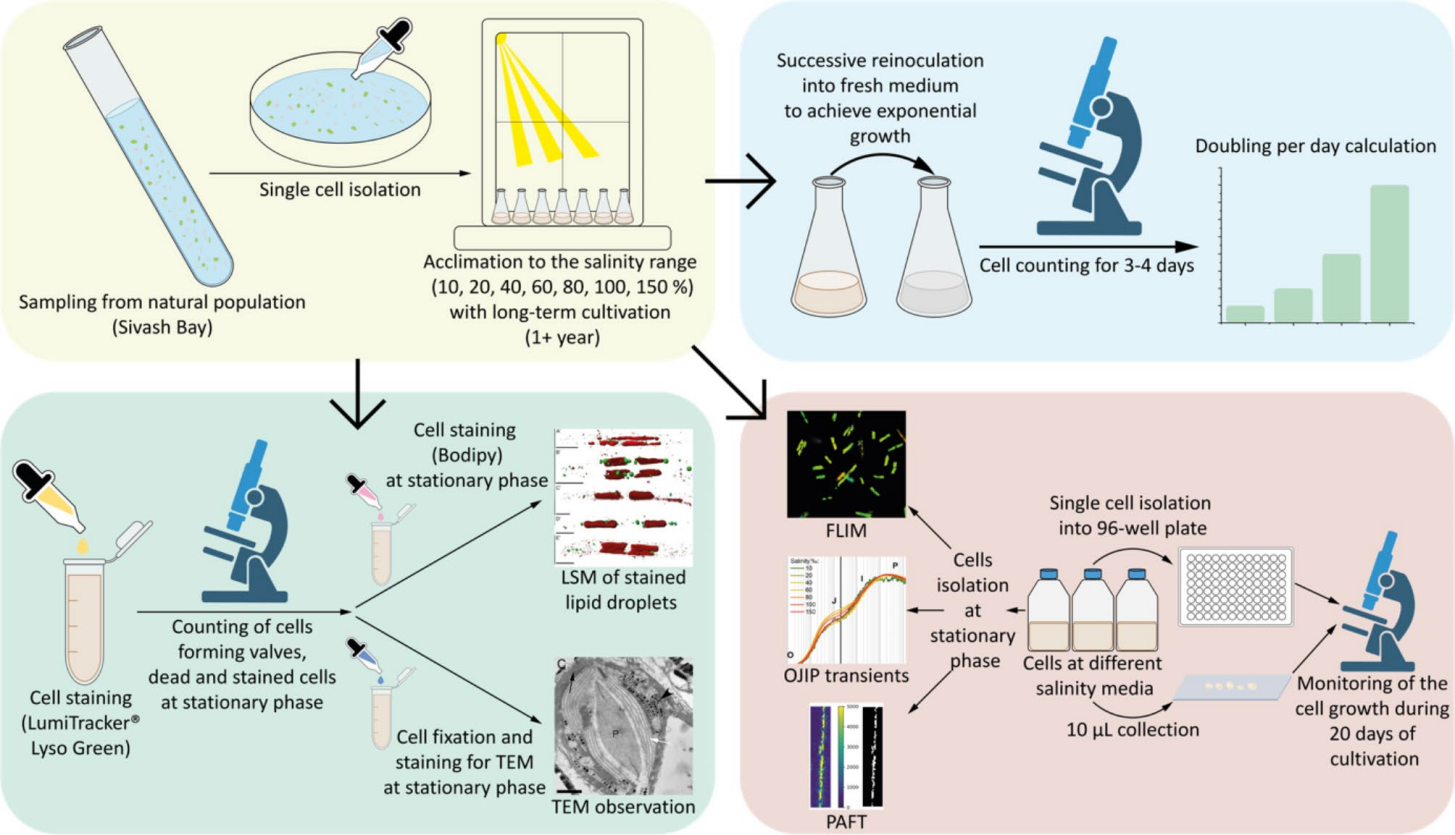
Flowchart of the experimental procedures for diatom analysis. The background color matches the background color of the results presented in Fig. 3.

Figure 2 presents a flowchart of the experimental procedures, beginning with diatom sampling from natural populations. The subsequent steps include cell isolation, acclimation to different salinity ranges, cell counting to determine growth rates and monitoring of cell growth. The diagram also illustrates the various experimental analyses carried out using different setups, including LSM, TEM, FLIM, chlorophyll fluorescence induction, and PAFT. All procedures and measurements are described in the materials and methods section.

**Fig. 3:**
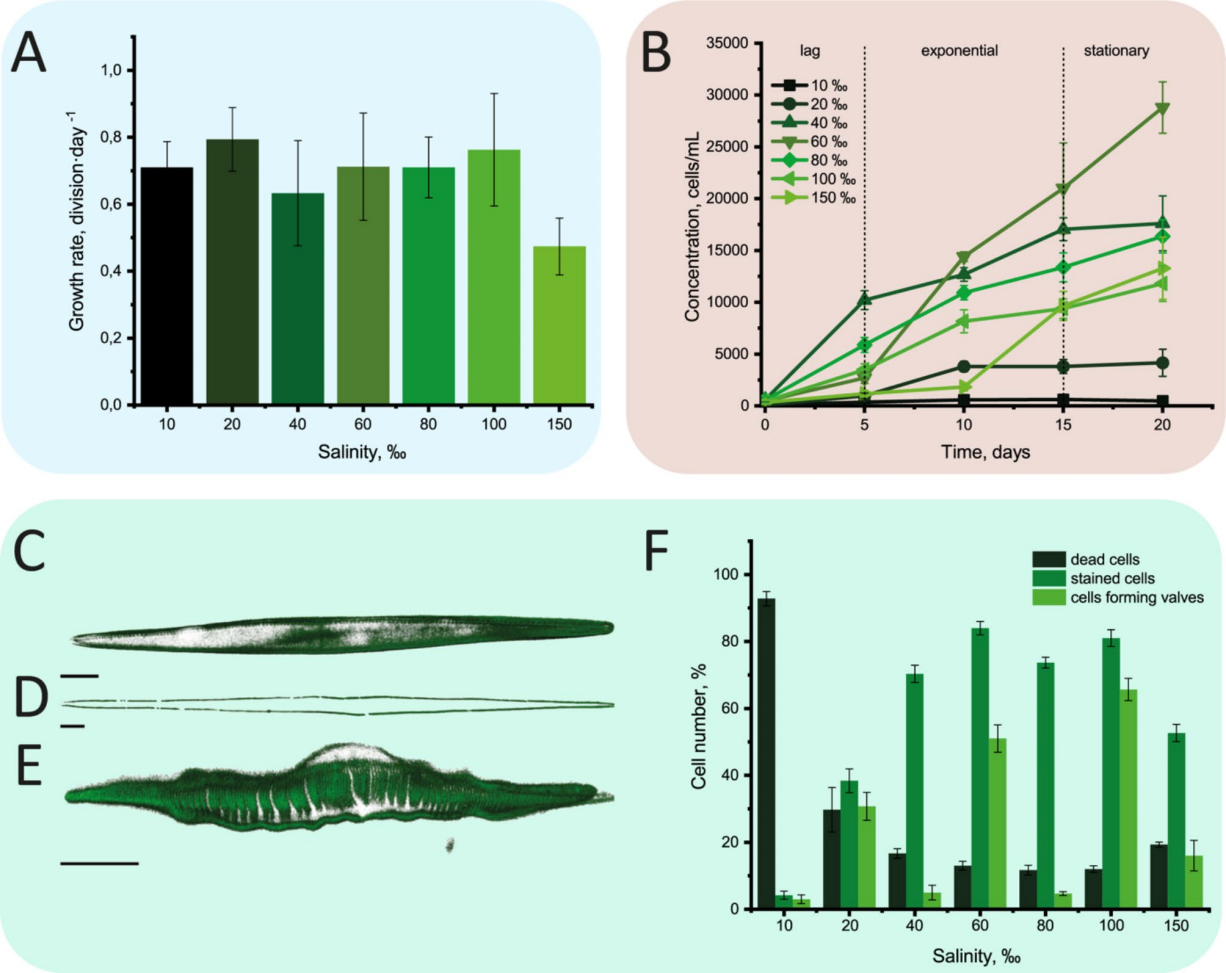
(**A**) Cell division rate of *Nitzschia* sp. in environments with different salinities in the first three days of cultivation. (**B**) Growth of *Nitzschia* sp. in medium with different salinities during 20 days of cultivation with indicated growth phases. Staining of *Nitzschia* sp. forming frustules using LumiTracker Lyso Green at stationary phase: (**C–E**) laser scanning microscopy, 3D-reconstruction. (**C**) valve; (**D**) girdle band; (**E**) valve morphology anomaly; (**F**) The proportion of dead cells of *Nitzschia* sp. and cells with LumiTracker Lyso Green staining at different salinities in the environment. Scale – 10 μm. The values are represented as the mean ± standard deviation.

Figure 3A illustrates the relationship between the division rate and salinity levels at specified increments of 10, 20, 40, 60, 80, 100 and 150‰. The findings suggest a relatively consistent tolerance to all tested salinity levels ranging from 10 to 150‰. For strains grown at salinities of 10 and 20‰, active growth was characteristic during the first week of cultivation; however, their viability during long-term cultivation was reduced. The growth curves depicted in Fig. 3B were recorded over a 20-day cultivation period of diatoms. The salinities of 10 and 20‰ show minimal growth, with cell concentrations remaining very low throughout the experiment. After 20 days, both 10‰ and 20‰ exhibit little to no increase in cell concentration, suggesting that these low salinities do not support sustainable growth for this strain during long-term cultivation. At 40‰, we observe a moderate growth rate. While this salinity allows for some cell proliferation, the overall concentration remains lower than that observed at higher salinities, indicating that 40‰ is tolerated but not optimal for maximum growth. The salinities of 60 and 80‰ display the highest growth rates and cell concentrations. Cultures at these salinities reach significantly higher cell concentrations by the end of the experiment, highlighting an optimal range for growth between 60‰ and 80‰. Although 100‰ shows a steady growth rate, it remains lower than 60‰ and 80‰. While cells can grow at this salinity, the rate and maximum concentration are not as high as those observed in the optimal range. At 150‰, growth is limited, and the final cell concentration is much lower than at 60‰ or 80‰. Supplementary Figure S1 shows the 20-day monitoring of diatoms at different salinities following the isolation of single cells in microvolumes. Supplementary Figure S2 presents a graph showing the relationship between division rate and salinity, based on data collected from the first to the tenth day of this experiment.

The staining of forming valves and lipid droplets was carried out at the stationary phase of culture growth. Following a four-week cultivation period, Lumitracker Lyso Green staining indicated that silicon assimilation by diatom cells occurred within environments exhibiting salinities ranging from 10 to 150‰ (Fig. 3C). The lowest number of stained valves was found at 10 and 20‰. Predominantly, cells cultured at salinities of 40 and 80‰ exhibited formation of girdle bands only (Fig. 3D, F). Notably, among the cells cultivated at a salinity of 60‰, anomalies in valve structure were observed, with stained valves exhibiting convex part (Fig. 3E). The number of cells assimilating silicon from the environment was minimal at a salinity of 10‰, where dead cells accounted for up to 92% at the stationary phase. At a salinity of 20‰, cell mortality was also elevated, with approximately 30% of cells being dead (Fig. 3F). Visualization of the cells after Lumitracker staining as 3D scatter plot made using the Python Matplotlib library is shown in Supplementary Fig. S3. SEM images of cleaned *Nitzschia* sp. frustules are shown in Supplementary Fig. S4.

**Fig. 4:**
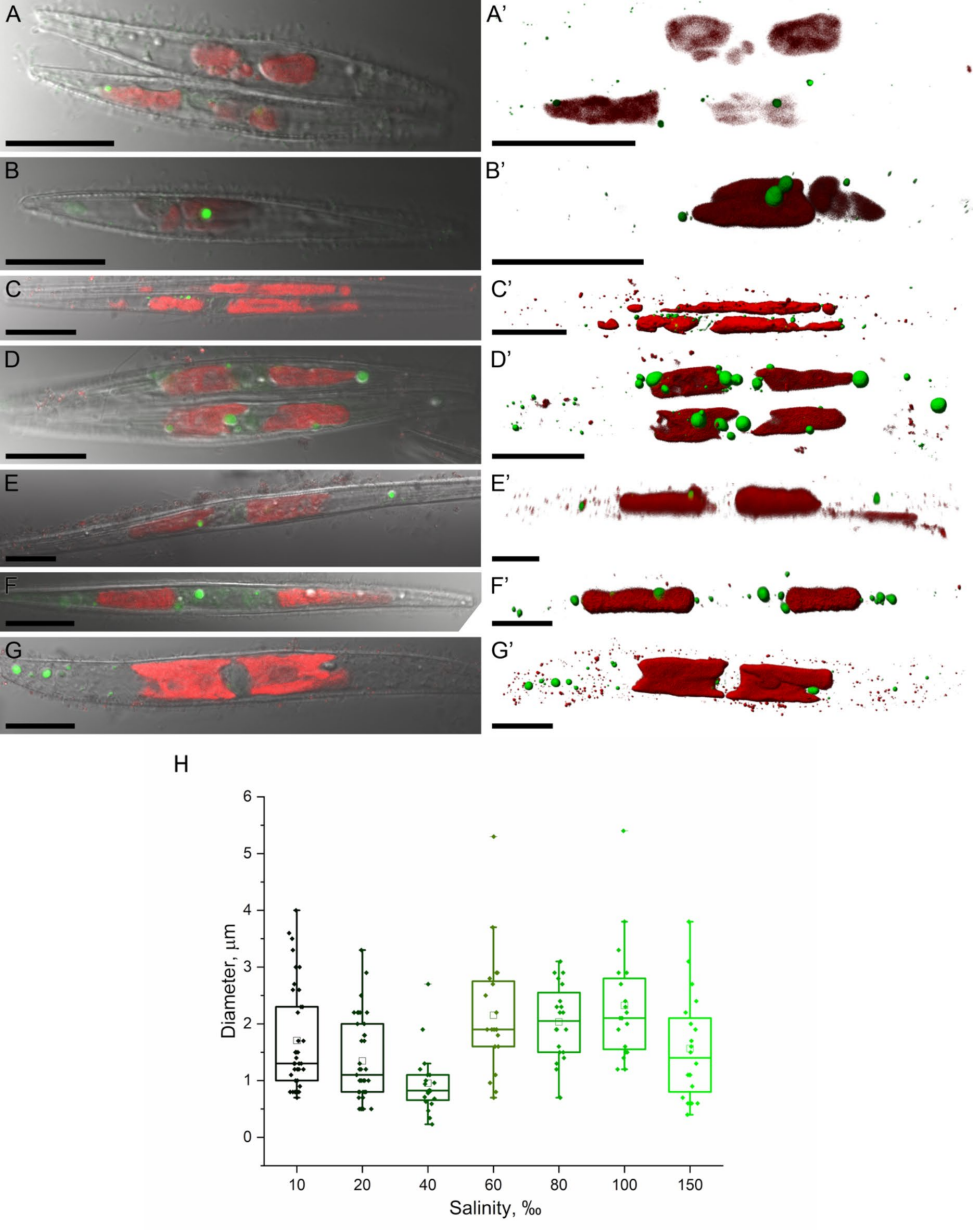
Lipid droplets in *Nitzschia* sp. cells. (**A-E’**) LSM, (**A-E**) optical section; (**A’-E’**) 3D-reconstruction; green – staining of lipid droplets with BDP 505/515, red – autofluorescence of chloroplasts. (**A-A’**) 10‰; (**B-B’**) 20‰; (**C-C’**) 40‰; (**D-D’**) 60‰; (**E-E’**) 80‰, (**F-F’**) 100‰, (**G-G’**) 150‰. (**H**) sizes of lipid droplets in *Nitzschia* sp. cells grown at different salinities. Scale – 20 μm.

Lipid droplets staining revealed that the accumulation of neutral lipids occurs in living cells within a salinity range from 10 to 150‰ (Fig. 4), while cells grown at a salinity of 40‰ exhibited lipid inclusions of a smaller diameter. Since the distribution of lipid droplet sizes was not normal for cells grown at different salinities, the Kruskal-Wallis test was used followed by multiple comparison of mean values as post-hoc analysis. P-values were significant, the mean value of lipid droplet sizes was significantly different in cells grown at 40‰ (Supplementary Table S1).

**Fig. 5:**
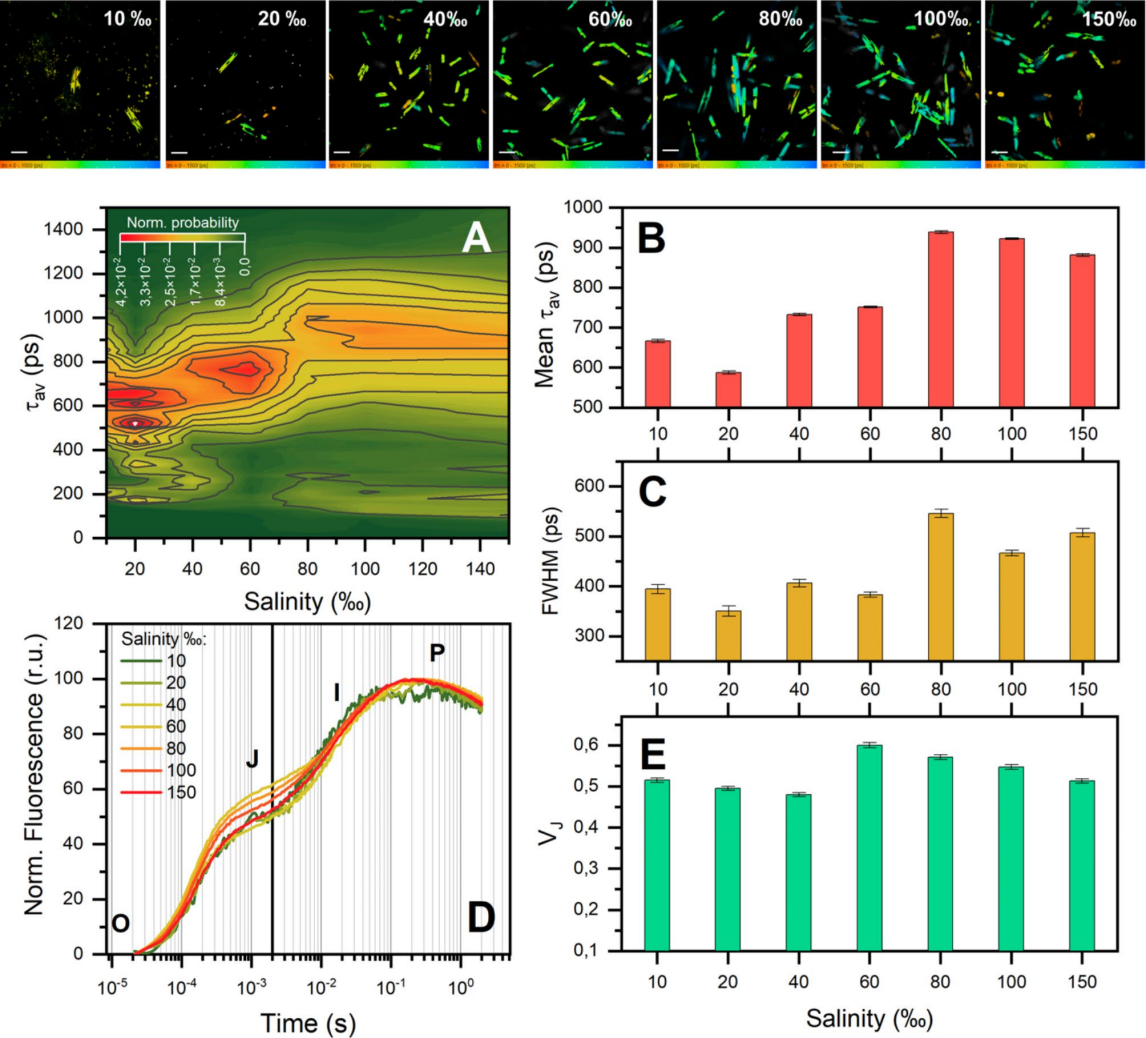
Fluorescence analysis of *Nitzschia* sp. cells using FLIM (top row of images - scale – 100 μm, panels **A**-**C**)) and chlorophyll fluorescence induction (panels **D**-**E**). (**A**) Distribution of mean chlorophyll fluorescence lifetime at different salinity values. Cell fluorescence was excited at 640 nm and detected in the channel over 660 nm. The main peak of the distribution was approximated by a Gaussian to determine the position of the maximum (**B**) and full width at half maximum (**C**) of the distribution of mean lifetimes. (**D**) Characteristic chlorophyll fluorescence induction curves of cells at different salinity values. Fluorescence intensity values at the “O” level (F_0_) are normalized to zero, while the fluorescence intensity values at the maximal “P” level (F_m_) are normalized to 100 for clarity. (**E**) Dependence of the index value V_j_ = (F_j_ - F_0_) / (F_m_ - F_0_) on salinity. The values are represented as the mean ± standard deviation.

The results represented in Fig. 5 give us a detailed view of the physiological changes in *Nitzschia* sp. cells under varying salinity conditions, specifically focusing on the fluorescence characteristics which are tied closely to photosynthetic activity.

To enhance the robustness of the data derived from various *Nitzschia* sp. cells, the cell suspensions grown under different salinity conditions were concentrated via centrifugation and examined across ten separate fields. The mean fluorescence lifetimes spanned between 570 and 950 picoseconds (Fig. 5B) in the main peak of the distribution. Only at 60‰ salinity we did not observe an additional minor distribution peak in the region of about 200 ps, probably indicating less functional heterogeneity of the cell population under these conditions. The fluorescence lifetime was shortest at a salinity of 20‰ and reached its peak at 80‰ (Fig. 5B, C).

The fluorescence transients recorded on dark adapted plants and algae exposed to the strong light are characterized by a multiphasic rise from the minimum (O) to the maximum (P) level via two intermediate steps J and I (Fig. 5D). There’s a notable fluctuation in V_j_ with different salinity levels, indicating the cellular response to light and the efficiency of the reduction of primary quinone acceptor of electrons at photosystem II (Fig. 5E). On the other hand, no changes were detected in the F_v_/F_m_ parameter.

**Fig. 6:**
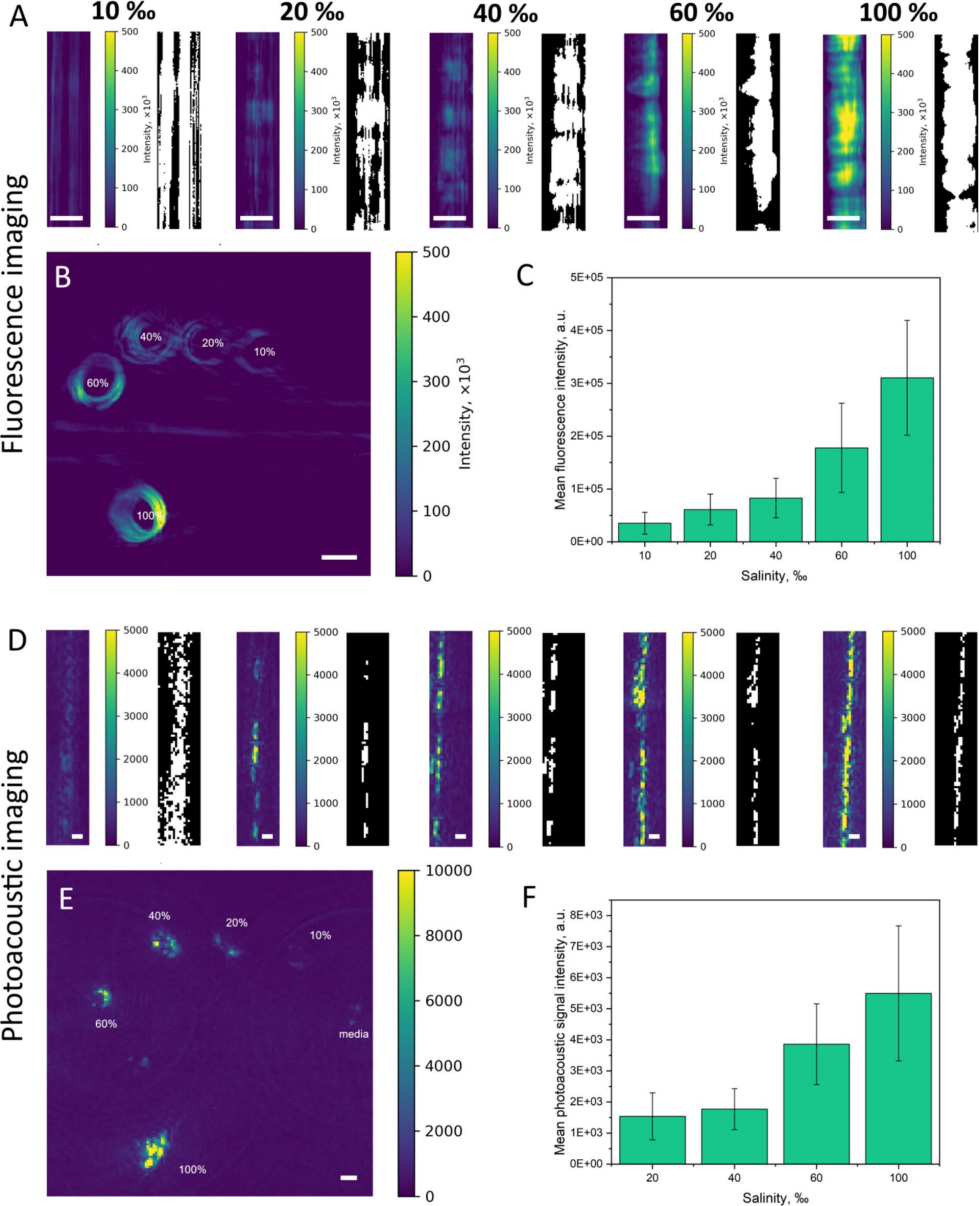
Fluorescence and photoacoustic imaging of *Nitzschia* sp. under varying salinity conditions. Fluorescence signal (excitation 460 nm, emission 646–795 nm) in: (**A**) longitudinal projections, (**B**) cross-sections of tubes containing *Nitzschia* sp. (**C**) The dependence of mean fluorescence intensity on salinity. Photoacoustic images (excitation 680 nm) of: (**D**) longitudinal projections, (**E**) cross-sections of tubes containing *Nitzschia* sp. at varying salinities. (**F**) The dependence of mean photoacoustic signal intensity with salinity. The black and white images represent longitudinal projections with an overlaid mask used to calculate mean signal intensity. Scale – 0.5 mm. The values are represented as the mean ± standard deviation.

Figure 6 illustrates the signals collected from diatoms at varying salinity levels using a PAFT imaging platform. Figure 6A depicts fluorescence imaging in longitudinal projections of *Nitzschia* sp. across salinity levels from 10 to 100‰. These projections highlight how the fluorescence signal changes along the length of the cell, with black and white images emphasizing specific areas of interest through the application of a mask using the Otsu method that assists in calculating the mean signal intensity. Figure 6B presents cross-sectional fluorescence imaging of *Nitzschia* sp. within silica tubes at various salinities, offering a complementary perspective to the longitudinal views and demonstrating the spatial distribution and concentration of fluorescent compounds within the diatom cells. As depicted in Fig. 6C, the mean fluorescence intensity increases with the increase in salinity. Figure 6D and E show photoacoustic imaging in longitudinal projections and cross-sections of silica tubes containing diatoms, respectively, revealing how photoacoustic signal intensity varies with salinity. The graph in Fig. 6F indicates a general increase in photoacoustic signal intensity with higher salinity levels. The correlation matrix was calculated using pandas.DataFrame.corr with the Pearson method. The resulting correlation coefficient is 0.98, indicating a direct relationship between the optoacoustic and fluorescence intensity values.

Wavelength shifts can occur between fluorescent emission, absorption, and the resulting photoacoustic signal. Discrepancies between optoacoustic and absorption spectra, as highlighted in the study by Werner et al.^36^, can often be attributed to factors such as the aggregation state of the dyes or the isomeric state of the chromophores. However, the strong correlation observed in this study suggests that the optoacoustic signal is primarily influenced by the same chromophore concentration as the fluorescence signal. Notably, while both fluorescence and optoacoustic signal intensities are proportional to the extinction coefficient of the chromophore, the fluorescence signal scales with the quantum yield (QY), whereas the optoacoustic signal strength is proportional to (1-QY)^37^. This inherent relationship between the two signals accounts for the high correlation coefficient of 0.98 observed in our study.

**Fig. 7:**
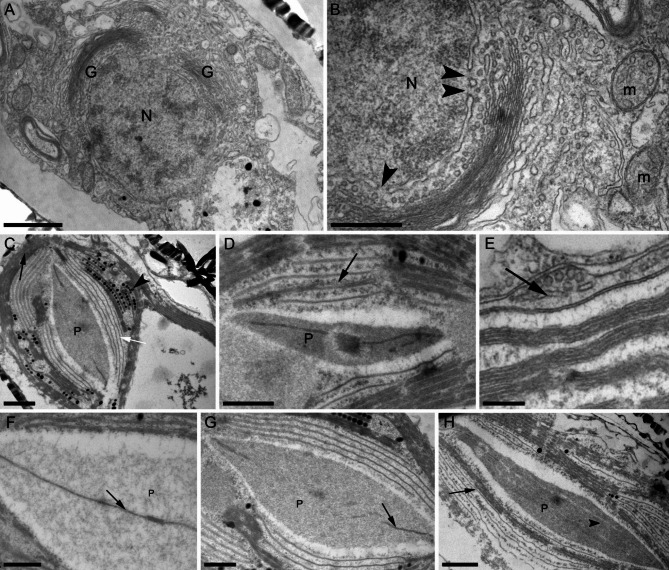
Ultrastructure of *Nitzschia* sp. cells (TEM). (**A**) section through the nucleus surrounded by dictyosomes; (**B**) enlarged fragment of Fig. 5A, arrowheads – nuclear pores; (**C**) section through the chloroplast, arrowheads – plastoglobules, black arrow indicates densely packed thylakoids, white arrow – areas with single thylakoids; (**D**) pyrenoid intersected by a complex lamella; (**E**) periplastidial reticulum (arrow); (**F-H**) sections of pyrenoids with different variants of lamella structure. Legend: G – dictyosomes; m – mitochondria; N – core; P – pyrenoid. Scale: A, C, H – 1 μm; B, D, F, G – 500 nm; E – 200 nm.

Transmission electron microscopy analysis revealed that the cells of the examined strains, cultivated across various salinity levels, have common structural features (Fig. 7). Ultra-thin sections of cells grown at salinities from 20 to 150‰ were successfully obtained. Notably, for cells grown at 20‰, no more than four live cells were found among 40 on the sections. The nucleus is located in the center of the cell and is surrounded by a ring of dictyosomes (Fig. 7A). The nuclear pores face the dictyosomes (Fig. 7B). The chloroplast has lobes, clearly visible on sections near the pyrenoid (Fig. 7C). The pyrenoid may be intersected by a lamella with a complex structure in the center of the pyrenoid; the lamella may appear as a semicircle in sections or be discontinuous (Fig. 7C, D, F-H). Plastoglobules are located in the stroma of the chloroplast as clusters, or as single droplets between stacks of thylakoids (Fig. 7C). Thylakoids are packed in stacks of three, rarely four. Next to the pyrenoid, the thylakoids are arranged in a region of single lamellae (Fig. 7C). The periplastidial reticulum can be either facing the cytoplasm or adjacent to the plasmalemma (Fig. 7E). Mitochondria are round, less often elongated, and located at the cytoplasm periphery (Fig. 7A, B).

**Fig. 8:**
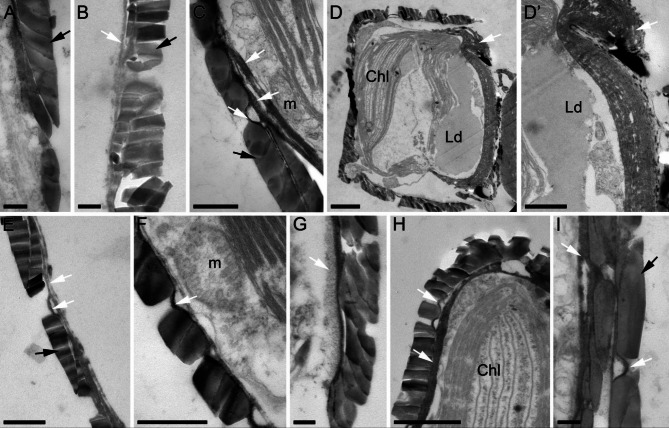
Features of diatotepum during *Nitzschia* sp. cell growth in an environment with salinities of 20 (**A**), 40 (**B**, **C**), 60 (**D**, **D’**), 80 (**E**, **F**), 100 (**G**, **H**) and 150‰ (**I**). Black arrows – girdle bands; white arrows – layers of diatotepum. Legend: Chl – chloroplasts; Ld – lipid droplets; m – mitochondria. Scale: A, B, G, I – 200 nm, C, D’, E, F – 500 nm, D, H – 1 μm.

A characteristic feature of the studied strains is the differences in the morphology of the polysaccharide layer, diatotepum, located between the frustule and the plasmalemma (Fig. 8). In cells grown at 20‰, this layer is usually almost indistinguishable, and at 40‰, it looks as a thin layer pressed against the frustule (Fig. 8A, B). With salinity increasing, the number of cells with a thickened diatotepum increases; the most substantial thickening, comparable in thickness to the shell, was observed in cells at 60‰ (Fig. 8D, D’). Diatotepum often has several clearly visible layers (Fig. 8C) and the size may vary in the layers adjacent to both the valve and the girdle bands (Fig. 8A, B, E, G, H, I).

## Discussion

*Nitzschia* sp. exhibited a relatively steady division rate across a broad spectrum of salinities, ranging from 10 to 150‰ (Fig. 3A). However, the data in Fig. 3B clearly show that cells grown at 10‰ exhibit a lower growth rate, and Fig. 3F highlights a high proportion of dead cells in accumulative cultures compared to other salinities. Despite this, cultures that had been acclimated to different salinities for a year and were inoculated into the same salinities during their exponential phase continued to grow without experiencing a lag phase, showing similar growth rate trends across all salinities, as illustrated in Fig. 3A. Therefore, while the growth dynamics differ in accumulative cultures, the long-term acclimation results in a comparable growth rate across the salinity spectrum, complicating the identification of an optimal salinity for vegetative growth.

This extensive halotolerance enables its proliferation across diverse ecotopes, accommodating environments with either exceptionally high or fluctuating salinity levels. Consequently, this adaptability allows *Nitzschia* sp. to inhabit a variety of ecological niches, including salt lakes, salt marshes, and estuaries. The observed division rates of *Nitzschia* sp. (Fig. 3A) do not qualify as elevated; rather, they align with the median division rates reported for numerous pennate diatoms under standard cultivation conditions that do not specifically promote accelerated growth (e.g., moderate illumination, temperature, and nutrient availability)^38^. Nevertheless, the species’ extensive halotolerance presents a considerable advantage for biotechnological applications, particularly in the utilization of highly saline waters, which are otherwise of limited utility. Moreover, its capacity to endure elevated salinity levels could serve as a strategy to safeguard *Nitzschia* sp. cultures from potential contaminants that are adversely affected by such salinity conditions^39,40^. Therefore, it is evident that physiological adaptations enable *Nitzschia* sp. to withstand substantial salinity variations.

Figure 3B shows the change in cell concentration of *Nitzschia* sp. over 20 days of cultivation at different salinity levels (10, 20, 40, 60, 80, 100 and 150‰). During the lag phase, there is little to no increase in cell numbers, which may be due to the algae adapting to the new conditions. After acclimation, cells enter the exponential phase, where rapid growth is observed. This phase is followed by the stationary phase, where there is a slow-down of cell division, likely due to the depletion of nutrients, reduced pH levels, shifts in bacterial community composition, or accumulation of metabolites in the medium^21,41^. The graph indicates that algae grow best at 60‰ salinity, although the transition to the stationary phase of growth in varying salinity environments occurs at disparate intervals (Fig. 3B).

Certain fluorescent vital dyes, known to accumulate in organelles exhibiting acidic pH, can be integrated into the developing valves and girdle bands of diatoms. This integration facilitates the assessment of diatom cell viability and enables the visualization of morphogenetic characteristics associated with valves and girdle bands^42–44^. Laser scanning microscopy after staining revealed that, upon reaching the stationary phase of growth after a four-week cultivation period, cells persistently engaged in the synthesis of valves subsequent to division (Fig. 3C) and in the formation of girdle bands (Fig. 3D). Significantly, this approach facilitated the identification of atypical valve anomalies within cells exposed to a salinity concentration of 60‰ (Fig. 3E), which remained undetected in assessments conducted via scanning electron microscopy.

In microalgal cells, lipid droplet accumulation is observed during the stationary phase of growth under culture conditions, particularly in response to nutrient scarcity in the environment^45,46^. The effect of increased salinity on lipid metabolism in green microalgae has been studied most thoroughly. It is shown that increased salinity and salt stress lead to starch-to-lipid biosynthesis in alga *Chlamydomonas*^47–49^. It is assumed that the accumulation of neutral lipids contributes to membrane integrity, which can be disrupted due to osmotic imbalance^50^. The analysis of the obtained results suggests that higher salinity activates the lipid metabolism mechanisms, enhancing the viability of cells at 60, 80, 100 and 150‰. Statistical analysis allows us to single out *Nitzschia* sp. cells at 40‰, since at this salinity smaller lipid droplets were observed, which may indicate fewer active processes of lipid synthesis and formation of lipid droplets. The observed tendency to decrease the size of lipid droplets suggests that at low salinities (40‰ and below), the accumulation of lipids in the cytoplasm of the studied species occurs more slowly due to the slow synthesis of fatty acids. Based on the growth dynamics of the analyzed strains (Fig. 3A, B), along with observations of valve formation post-division (Fig. 3F) and lipid inclusion accumulation (Fig. 4) within *Nitzschia* sp. cells, it is evident that lipid metabolism exhibits deceleration at salinity levels of 40, 80, and 150‰. This contrasts with the metabolic activity observed at salinities of 60 and 100‰, where a relatively enhanced rate of lipid accumulation and valve formation is noted.

The results of the study were expected to show a smooth increase and decrease in the size of lipid droplets and the number of stained valves, however, this did not happen due to the fact that several cell lines of a monoclonal strain acclimated to different salinities for a long time were used in the study. It is known that strains of diatoms during growth in artificial conditions can have different cell morphology during cultivation. After numerous divisions, the cell size decreases and frustules with morphological anomalies appear as a result of disturbances in valve morphogenesis by an unknown mechanism^51,52^. Long-term maintenance of the culture at different salinities is the cause of different degrees of changes not only in lipid metabolism, but also in the synthesis of the polysaccharide layer. Other changes are also very likely, leading to the fact that the onset of the stationary phase in the culture occurs at different times in strains acclimated to different salinities. Since the strain was isolated from an environment with a wide range of salinity, it is difficult to assume which environment is the most natural and optimal for the survival of the species in a natural population. It is obvious that the isolated strain is adapted specifically to environmental changes.

The average fluorescence lifetimes ranged from 570 to 950 picoseconds (Fig. 5B). This range aligns with findings from study by Wu et al.^53^, which demonstrated that the fluorescence lifetime peak for *Thalassiosira weissflogii* in control conditions was around 760 ps, increasing to approximately 940 ps after 72 h of exposure to 1.1 µM Hg(II). The observed increase in fluorescence lifetimes with higher salinity, as illustrated in the heatmap (Fig. 5A), could imply stress-induced modifications within the cells of *Nitzschia* sp. However, the staining of the forming frustules, which characterizes cell viability, as well as the resilience of strains grown at higher salinity, suggests that modifications of the photosynthesizing complexes for this species allow for more effective adaptation to conditions of high salinity. Notably, longer fluorescence lifetimes may reflect impaired energy transfer efficiency within the photosystems, possibly due to alterations in chlorophyll-protein complexes under hyperosmotic stress conditions. A short fluorescence lifetime often means that energy absorbed by chlorophyll molecules is rapidly transferred to the photosystems (specifically Photosystem II), which can be indicative of active photosynthesis. This suggests that the absorbed light energy is effectively being used for photochemistry rather than being re-emitted as fluorescence. However, a reduced fluorescence lifetime can also be a sign of non-photochemical quenching (NPQ), protective mechanism microalgae use to dissipate excess energy as heat. Short fluorescence lifetimes can serve as indicators of a healthy and active photosynthetic process in microalgae under optimal conditions. But, if stress is applied (like in our cases varying salinity levels), a shortened lifetime may also indicate protective responses.

The observation that diatom algae grown at 20‰ salinity exhibit the shortest fluorescence lifetime, even with around 30% dead cells at the stationary phase, suggests a complex interaction between stress responses and the state of the photosynthetic apparatus in the surviving cells. At 20‰ salinity, only the most resilient diatom cells may survive, and these cells could be highly stressed. In response, the surviving cells might increase their NPQ capacity, leading to shorter fluorescence lifetimes. Salinity stress generally affects the photosynthetic apparatus, potentially leading to photodamage or impaired reaction center activity. Surviving cells could be activating quenching mechanisms more intensely than under normal conditions. These mechanisms shorten the fluorescence lifetime, as energy is quickly quenched to prevent damage. In the stationary phase under high salinity, the diatom culture may be heterogeneous, with a mix of dead, damaged, and stressed but still photosynthetically active cells. Our results (Fig. 3F) indicate that, although 30% of the cells are dead, another 30% are actively forming valves during the stationary phase. The fluorescence measurement typically captures the average response of the live cell population rather than the dead cells (since dead cells exhibit minimal or no fluorescence). Thus, the measured fluorescence lifetime reflects only the live cells, which are likely maximizing their quenching efficiency to cope with the salinity. According to the study by Pavlinska et al.^54^, shifts in fluorescence lifetimes under environmental stress indicate adjustments in the photosynthetic apparatus to maintain functionality. Our data suggest that similar changes may also enhance cell survival in conditions of increased salinity.

Furthermore, the broadening FWHM suggests that these changes are not uniform across the cell population, which may point to different adaptive strategies and reflect the complexity of internal physicochemical processes within the cells (Fig. 5C)^32^. Such differences could stem from a variety of factors, including the composition and structure of the cell membranes, variations in metabolic enzyme activity levels, set of photosynthetic pigments and the unique physiological properties of individual cells. Additionally, fluorescence mechanisms might respond to environmental shifts, such as changes in salt concentration, which could alter the internal cell microenvironment and impact the chemical reactions underpinning fluorescence^55^. As salinity increases, the ionic strength of the medium changes, which can alter the microenvironment around the fluorescent molecules within the diatoms. This alteration can lead to changes in the molecular interactions and the photophysical properties of the fluorophores, potentially stabilizing the excited state and resulting in longer fluorescence lifetimes. Besides, salinity changes can cause shifts in the pH of the nutrient medium. Since the fluorescence properties of many fluorophores are pH-sensitive, such shifts could directly affect their fluorescence lifetime^56^.

The changes in OJIP transients and the Vj index value indicate salt-induced partial limitation of electron transfer from Qa to the plastoquinone (PQ) pool. The initial level “O” corresponds to chlorophyll fluorescence intensity at 50 µs with open reaction centers (RCs) of PSII (F_0_) when all Qa are oxidized. The O-J phase (Fig. 5D) corresponds to a major reduction of the PSII acceptor side, while the following JIP rise has been suggested to reflect the sequential reduction of the PQ pool and electron carriers beyond the PQ pool. Peak P corresponds to maximal recorded fluorescence intensity with closed RCs of PSII (F_m_) when all Qa are reduced^33,57,58^.

OJIP pattern can differ between various algae classes^59^. In diatom algae the I step is fully absent (or significantly reduced) in the fluorescence transients unlike green algae^60^. Such a shape can be due to the effect of membrane potential which enhances fluorescence yield during the J-I phase^60^. In OJIP transients, an increase in the amplitude of the O-J phase (parameter V_j_) was observed in the range of 60–100‰, with a maximum increase at 60‰ (Fig. 5E), while no changes were detected in the F_v_/F_m_ parameter. The increase in the amplitude of the O-J phase can be due to a number of factors, including PSII heterogeneity (PSIIα and PSIIβ centers), the Qb-reducing / non-reducing heterogeneity of PSII, damage on the donor side (peak K at ~ 300 µs), and the influence of the PQ pool dark reduction (rise of F_o_)^61^. An increase in the total number of closed RCs incapable of re-oxidation of Qa and reduction of the secondary electron acceptor Qb leads to an increase in the amplitude of the O-J phase. The evidence is the action of photosynthetic electron transport inhibitor diuron, which displaces Qb from its binding site on the D1 protein of the PSII RC. Diuron disrupts the electron transport between Qa and Qb, and leads to reduction of OJIP curves at J step^61^. In this study, the increase in the amplitude of the O-J phase may be due to the partial limitation of electron transfer from Qa to the PQ pool by a moderately enhanced basal ion flux at increased NaCl concentrations as was previously shown^62,63^.

Using a combination of fluorescence and photoacoustic imaging with a PAFT imaging platform (Supplementary Fig. S5) to examine *Nitzschia* sp., we observed a pronounced increase in both signal intensities corresponding to elevated salinity levels (Fig. 6C, F). This enhancement can be largely attributed to the dynamics of chromophore concentrations within the diatoms, primarily focusing on the role of chlorophyll *a*, the predominant chromophore in these microalgae. The photoacoustic effect is driven by the absorption of pulsed laser light by cellular chromophores, followed by non-radiative relaxation processes that generate acoustic waves^32,64^. For the measurements, a laser line at 680 nm was used to specifically excite chlorophyll *a*. This wavelength is strongly absorbed by chlorophyll *a*, ensuring a clear photoacoustic signal that allows for effective imaging of the pigment distribution within the diatom (Supplementary Fig. S6). There is a good correspondance between mean photoacoustic signal excited at 680 nm and absorbance values at 680 nm (Supplementary Fig. S7). The increase in photoacoustic signal with salinity could suggest a modulation in the concentration or the optical properties of chlorophyll *a*, potentially as a physiological response to osmotic stress induced by the saline environment. As explained before, salinity fluctuations are known to influence photosynthetic organisms by altering their internal ionic balance and potentially triggering osmoprotective mechanisms, which include changes in chlorophyll concentration^65^. Furthermore, the interaction between chlorophyll *a* and other accessory pigments like carotenoids, which also respond to environmental stress, could compound the effects on the photoacoustic signals^32^. These pigments not only contribute to light absorption but also play a critical role in photoprotection mechanisms under stress conditions^66^. Their altered abundance or spatial arrangement relative to chlorophyll *a* under varying salinity could modify the overall optical absorption properties of the diatoms, thereby influencing the photoacoustic output. These results provide a basis for using photoacoustic imaging to assess the health and metabolic status of these vital aquatic organisms in their natural or altered habitats.

Additionally, the fluorescent signal was sequentially recorded using laser excitation at 460 nm with detection in the range of 646–795 nm, which also corresponds to emissions from chlorophyll *a* (Supplementary Fig. S8). The choice of 460 nm for excitation takes advantage of the absorption by accessory pigments (such as carotenoids) that transfer energy to chlorophyll *a*, ultimately allowing for efficient fluorescence. The detected fluorescence emission is then attributed to chlorophyll *a*, providing complementary information to the photoacoustic data. While fluorescence provides insight into the re-emission of absorbed light, photoacoustics reveals the optical absorption characteristics. Together, these techniques give a more complete picture of the optical properties of diatoms and their photosynthetic activity.

The ability to measure salinity levels in real time using non-invasive imaging techniques is important for monitoring water quality in marine and estuarine environments. This could help in detecting changes due to pollution or climate change and aid in maintaining healthy aquatic ecosystems. Furthermore, understanding the effect of salinity on diatom physiology can help optimize the conditions for diatom growth in bioreactors, maximizing productivity for applications such as CO_2_ sequestration, biofuel production, or biosilica extraction. Diatoms can also serve as biological sensors for detecting salinity changes in aquatic environments. By developing a system that uses photoacoustic and fluorescence imaging to monitor diatom response, a reliable biosensor for salinity can be constructed, which could play a role in the sustainable management of aquaculture systems and in assessing the impacts of climate change on marine biodiversity.

Previously, the ultrastructure of cells of species inhabiting environments with a broad salinity spectrum, had not been studied. The microscopy results showed that the cell structure of *Nitzschia* sp. corresponds to data on the diatom cytology^67–70^, however, it also has its own characteristics, in particular, different morphology of thylakoids in chloroplasts (Fig. 7). Thylakoids can be either single or have a usual for diatoms pack of three in a stack. In the region of the pyrenoid, the thylakoids are arranged more sparsely, which was also noted earlier for *Entomoneis* cf. *paludosa*^71^. Presumably, this structure may reflect the location of photosystem I and II enzymes in thylakoid membranes. It has already been shown that such segregation of photosystems I and II exists on the thylakoid domains in *Phaeodactylum tricornutum*, but does not have such a clear morphological difference^72^. Features of the lamella crossing the pyrenoid, such as stacked membranes (Fig. 7D), have not previously been described in other species and are likely to be a feature of this species.

Each component of the diatom frustule is encased in organic layers, among which the diatotepum is the most pronounced and observable^73,74^. This particular layer is situated in direct proximity to the frustule on one side while being adjacent to the plasmalemma on the opposite side. Pennate diatoms typically form a dense diatotepum, often almost completely covering the areola openings^43^. The ability to form a thickened layer of diatotepum is most likely characteristic of the studied species, regardless of the salinity of the environment; however, under certain conditions (for example, at a salinity of 60‰), such thickenings can lead to the formation of valves with an abnormal structure (Fig. 8D, D’). Interestingly, this morphology change does not associate with disturbances in the functioning of the cytoskeleton^71^, but is caused by the mechanical effect of the massive polysaccharide layer on the developing valve, which in the early stages of morphogenesis can be very plastic^75^. It is possible that in some cells, most often having a shorter length, the rate of diatotepum synthesis during valve morphogenesis becomes many times higher than the silica deposition and does not stop until exocytosis of the newly formed valve occurs. The data obtained suggest the existence of mechanisms regulating the formation of the polysaccharide layer during valve morphogenesis. Thus, changes in salinity may cause not only changes in silica density^76^, but also enhance the synthesis of the polysaccharide layer. Changes in ultrastructure and fluorescence dynamics due to salinity in the marine diatom *Nitzschia* sp. are summarized and depicted schematically in Supplementary Fig. S9.

## Conclusion

This study explored the effects of salinity on the marine diatom *Nitzschia* sp., across a wide range of salinities (10 to 150‰,), employing advanced photonic methods, including fluorescence lifetime imaging microscopy, photoacoustic and fluorescence tomography, and transmission electron microscopy. The results highlight *Nitzschia sp.* remarkable adaptability, though prolonged exposure to extreme salinities led to reduced resilience and cell mortality. At 20‰, the shortest fluorescence lifetime indicated enhanced non-photochemical quenching, a protective stress response. Growth and cell division were stable during the first week of cultivation, and ultrastructural analysis revealed no significant anomalies, suggesting preserved cellular integrity. At 40‰, early stress signs included smaller lipid droplets, but growth continued to increase, reflecting tolerance to moderate salinity. At 60‰, structural anomalies in valve morphology and increased lipid accumulation indicated metabolic stress, with fluorescence transients showing altered photosystem II functionality. Above 60‰, increased fluorescence lifetimes suggested reduced photochemical quenching efficiency, yet metabolic and structural adjustments allowed the strain to survive without major mortality.

By 100‰, growth rates declined, lipid droplet sizes varied, and salinity tolerance approached its upper limit of tolerance. At 150‰, severe physiological disruptions, including high cell mortality and silicon assimilation similar to 40‰, underscored the detrimental effects of extreme salinity. The increase in fluorescence and photoacoustic intensity with salinity levels highlights adaptive strategies to mitigate stress caused by changes in salinity. This study underscores the resilience of *Nitzschia sp.* to salinity fluctuations while identifying thresholds where physiological functions deteriorate. These findings pave the way for future research into leveraging marine microalgae for environmental monitoring and sustainable biotechnological applications.

## Experimental section

### Cultivation of diatoms and monitoring of their growth

Clones *Nitzschia* sp. were isolated from Sivash Bay (the Sea of Azov). At sampling sites in the natural population salinity levels were 40 and 202‰. The clones were gradually acclimatized to the slightly modified medium ESAW^77^ for a week and then maintained in a range of of 10, 20, 40, 60, 80, 100‰ during one year. To describe the division rate, cells were re-inoculated from exponentially growing cultures into fresh medium and counted on 15 fields of view with an inverted microscope Nib-100 (China) on days 1, 2 and 3 (cultures in two replicates) or 1, 3 and 4 (cultures in three replicates) after re-inoculation. To a high approximation, the population growth curves are described by the exponential equation:

1 $${N}_{t}={N}_{0}{e}^{r\cdot \Delta t}$$

where N_t_ and N_0_ – average cell number in the field of view at time t and initial time t_0_, ∆t – time interval between t and t_0_, r determines the specific rate of population growth (day^− 1^). For each salinity, mean r values and standard errors were calculated for five replicate experiments using the least squares method. To proceed to the division rate (K, divisions/day), the obtained r values were divided by ln2^78^.

To monitor culture growth over an extended period until reaching the stationary phase, two approaches were employed. First, single cells were inoculated into microvolumes in 96-well flat-bottom plates as previously described^79^. One cell per well (in at least 3 replicates) was inoculated in 200 µL of medium, and cells were counted every 5 days for 20 days using an inverted luminescent microscope NIB-FL (Lomo-MA, Russia). The second approach involved re-inoculating the cell culture into “T-25” cell culture flasks (Corning, USA) and counting cells from 10 µL drops on glass slides under the microscope (5 replicates) over 20 days at 5-day intervals across salinities of 10, 20, 40, 60, 80, 100, and 150‰.

### Laser scanning microscopy

Staining for laser scanning microscopy (LSM) was carried out after all strains were transferred to fresh medium at a concentration of 5–20 thousand cells/ml and cultured for four weeks.

To visualize lipid droplets, a solution of 0.5 mM BDP 505/515 (Lumiprobe, Russia) was added to the medium with cells to a final concentration of 2 µM. After 30 min, the cells were washed with a phosphate buffer (0.1 M, pH 7.4) and fixed with a 4% paraformaldehyde solution. The fluorescence of BDP 505/515 was excited by a 488 nm laser; emission was registered in the range of 476–556 nm. Autofluorescence of chloroplasts was excited by a 561 nm laser; emission was registered in the range of 650–723 nm. Three-dimensional reconstructions were obtained from 50 to 80 optical sections with a thickness of 0.3–0.5 μm along the z axis.

To describe the assimilation of silicon from the medium, as an indirect evidence of cell viability, LumiTracker Lyso Green (Lumiprobe, Russia) was used. The dye was added to the cells to a final concentration of 0.3 µM in the medium, cultured as usual for 1 day, and fixed with a 4% paraformaldehyde solution. After this, cells were washed with a phosphate buffer, mounted on glass slides in Mowiol medium (Sigma-Aldrich, USA) and examined using an LSM 710 laser scanning microscope (Zeiss, Germany) with a Plan-Apochromat 63x/1.40 Oil DIC M27 immersion lens (Zeiss, Germany). LumiTracker Lyso Green fluorescence was excited by a 488 nm laser, emission was registered in the range of 496–647 nm. Cells that had neither LumiTracker Lyso Green staining nor chloroplast autofluorescence were counted as dead. Three-dimensional reconstructions were obtained from 100 to 120 optical sections with a thickness of 0.1 μm along the z axis. Images were obtained and processed using ZEN 2010 software (Zeiss). All calculations were carried out among 100 randomly encountered cells in triplicate.

### Fluorescence lifetime imaging microscopy

Fluorescence lifetime imaging microscopy (FLIM) was performed in the time-correlated single- photon-counting (TCSPC) mode using the confocal system DCS-120 (Becker&Hickl, Berlin, Germany) installed on the Eclipse Ti2 (Nikon, Tokyo, Japan) microscope. Excitation was performed with a 640 nm picosecond laser BDS-SM-473-LS-101 (Becker&Hickl, Berlin, Germany) with a 30 ps duration impulse driven at 50 MHz repetition rate synchronized with hybrid detector HMP-100–40 C (Becker&Hickl, Berlin, Germany) via board SPC-150 (Becker&Hickl, Berlin, Germany). Cell fluorescence was detected in the channel over 660 nm.

### Fast chlorophyll *a* fluorescence (OJIP) transients

Fast chlorophyll *a* fluorescence measurements were carried out at room temperature with AquaPen-C AP-C 100 (Photon Systems Instruments, Czech Republic) instrument. The photosynthetic photon flux density (PPFD) and duration of the blue actinic flash (λ = 450 nm) were 3000 µmol photons m^[–2^ s^[–1^ and 2 s, respectively. Before measurements, the samples were adapted to the dark. The chlorophyll *a* fluorescence intensities values determined at 50 µs (F_0_ = O level), 2 ms (F_j_ = J step) and maximal recorded fluorescence intensity (F_m_ = P step) were used to calculate fluorescence parameters according to the equations proposed by Strasser et al.^80^ Relative variable fluorescence at the step J was calculated as V_j_ = (F_j_ - F_0_) / (F_m_ - F_0_) where (F_m_ − F_0_) = F_v_ – variable fluorescence at time t. Maximum quantum yield of PSII photochemistry (a probability of excitation energy trapping in PSII) was calculated as F_v_/F_m_ = (F_m_ - F_0_) / F_m_. The intensity of the chlorophyll _a_ fluorescence was recorded in arbitrary units, then transformed into relative units of the relative variable chlorophyll fluorescence by double normalization to the initial fluorescence level, F_0_, and to the maximum level, F_m_.

### Measurement of photoacoustic and fluorescence tomographies (PAFT)

TriTom (PhotoSound Inc, Houston, Texas, USA) is a setup that provides both measurement modalities: photoacoustic and fluorescent. The diatom suspensions grown at different salinity media were collected from cell culture flasks during the stationary phase and subsequently transported for PAFT measurements. First, the sample suspensions are placed in tubes, with both ends sealed. The tubes are then placed in a sample holder (see Supplementary Fig. S5, 1), which can accommodate up to 10 samples simultaneously. The sample holder is subsequently placed in an imaging chamber and immersed in a tank filled with bidistilled water. During the measurement, the sample holder rotates fully clockwise at a speed of 10° per second. The white light laser (PhotoSonus, Ekspla, Lithuania, with a wavelength range of 400–1300 nm) is used to apply laser pulses to the samples through laser waveguards (see Supplementary Fig. S5, 6). The photoacoustic signal is recorded using an array of photoacoustics transducers (see Supplementary Fig. S5, 4). To excite the optoacoustic signal, a 680 nm line was used with a pulse energy of 105 mJ (100%). The fluorescent signal was sequentially recorded using laser lines at 460 nm (41.3 mJ) with detection in the range of 646–795 nm. Detection was carried out using a CMOS camera, Dhyana 400BSI (Tucsen, Gaishan Town, China). The detection range is based on the optical filter used. Subsequently, 3D reconstruction of the fluorescence and photoacoustic data was performed. For groups of samples, 2D images of the cross-sectional maximum intensity projection were obtained. For each sample individually, a 2D image of the longitudinal maximum intensity projection was obtained. The average signal value and standard deviation for each sample were calculated by applying a mask to the longitudinal projections using the Otsu method, followed by analysis within a selected region of interest^81^.

### Transmission electron microscopy study of cell ultrastructure

To study the cell ultrastructure, we used the protocol proposed earlier^82^ with modifications. Cells were harvested with centrifuge MiniSpin (Eppendorf) and fixed with a mixture of glutaraldehyde (2.5%) and paraformaldehyde (1%) in the medium for 1 h, then the solution was changed to fresh 2.5% glutaraldehyde and 2% paraformaldehyde in phosphate buffer. After 2.5 h, the cells were washed with phosphate buffer solution and postfixed with 1% osmium oxide solution for 12 h. Further dehydration and impregnation in epoxy potting medium according to the previously accepted protocol^83^. Ultrathin sections were prepared using a Diatom diamond knife (Germany) on a Leica Ultracut ultramicrotome, stained with lead citrate and uranyl acetate, and examined using a Leo 906 E transmission electron microscope (Carl Zeiss, Jena, Germany) at 80 kV. Micrographs were obtained using a MegaView II digital camera (Olympus Soft Imaging Solutions, Münster, Germany).

### Statistical analysis

Statistical analyses were performed using Kruskal-Wallis test in STATISTIKA 7.0. software (Stat Soft Inc., Tulsa, OK, USA). Statistically significant differences were calculated at *p* ≤ 0.05. The results are displayed as the mean ± standard deviation (*n* = 3).

## Supplementary Information

Below is the link to the electronic supplementary material.

Supplementary Material 1

## Data Availability

All data generated during this study are included in this article.
