# Supplementary material for: Exploring salinity induced adaptations in marine diatoms using advanced photonic techniques
Source: Sci Rep. 2024 Dec 30;14:32007. doi: 10.1038/s41598-024-83640-9 (PMC11685788; doi:10.1038/s41598-024-83640-9)
Supplement: Supplementary file 1 — Supplementary Material 1 [file 41598_2024_83640_MOESM1_ESM.pdf]

Supplementary Information

Exploring salinity induced adaptations in marine diatoms using advanced photonic techniques

Julijana Cvjetinovic[1], Yekaterina D. Bedoshvili[1, 2], Nickolai A. Davidovich[1, 3], Eugene G. Maksimov[4], Ekaterina S. Prikhozhdenko[5], Daria A. Todorenko[4], Daria V. Bodunova[4], Olga I. Davidovich[3], Igor S. Sergeev[1], Dmitry A. Gorin[1]*

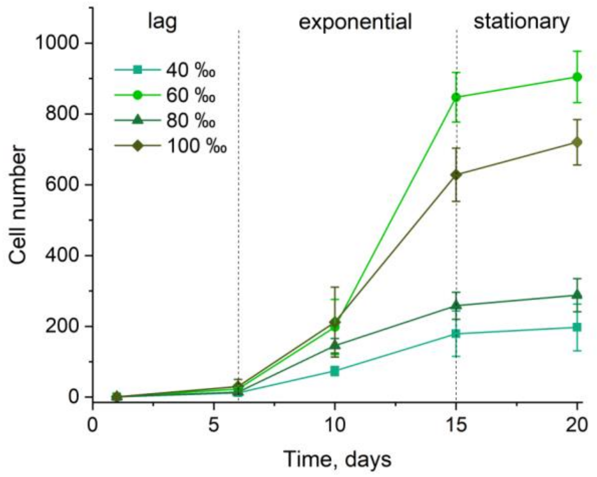

Figure S1. Growth of *Nitzschia* sp. in medium with different salinities during 20 days of cultivation after isolation of one single cell in microvolumes with indicated growth phases

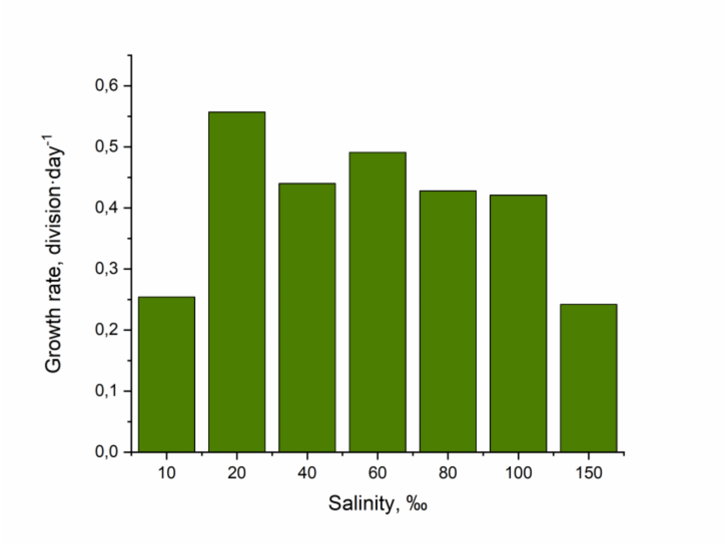

Figure S2. Growth rate of *Nitzschia* sp. calculated for the first ten days of the experiment based on the data provided in Figure 3B.

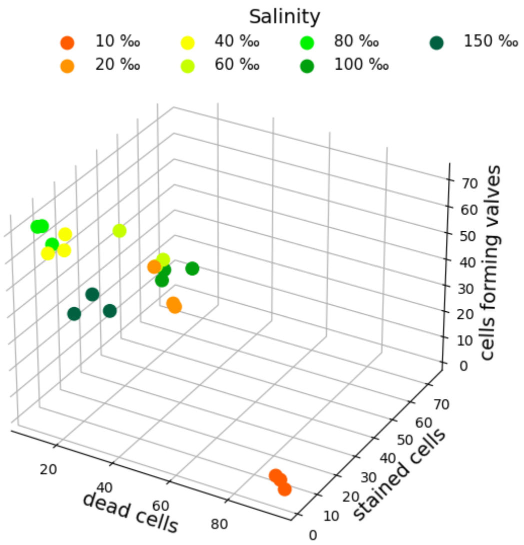

Figure S3. Visualization of the cells after Lumitracker staining as 3D scatter plot made using the Python Matplotlib library.

Table S1. Kruskal-Wallis test calculated with a p-value of 0.05.

‰	10 ‰ - R:91,603	20 ‰ - R:70,077	40 ‰ - R:43,375	60 ‰ - R:113,45	80 ‰ - R:117,10	100 ‰ - R:125,43	150 ‰ - R:81,925
**10**		1,000000	0,013993	1,000000	1,000000	0,357101	1,000000
**20**	1,000000		1,000000	0,046400	0,019034	0,001975	1,000000
**40**	0,013993	1,000000		0,000358	0,000127	0,000010	0,377812
**60**	1,000000	0,046400	0,000358		1,000000	1,000000	1,000000
**80**	1,000000	0,019034	0,000127	1,000000		1,000000	0,648375
**100**	0,357101	0,001975	0,000010	1,000000	1,000000		0,159486
**150**	1,000000	1,000000	0,377812	1,000000	0,648375	0,159486	

Cleaning of frustules for SEM

Diatom cells were harvested by centrifugation and incubated in three portions of 6% SDS at 95 °C on a water bath, 30 min each, with intermediate washing and pelleting in five changes of distilled water. The final pellet was treated with concentrated nitric acid at 95 °C on a water bath for 1 h, washed in three portions of 70% ethanol, and treated with concentrated hydrochloric acid for 24 h. The cleaned valves were washed in no less than five changes of distilled water and mounted on the SEM stubs and coated with gold in an SCD 004 sputter coater (Balzers) and viewed in a Quanta 200 scanning electron microscope (FEI, USA), as shown in Figure S4.

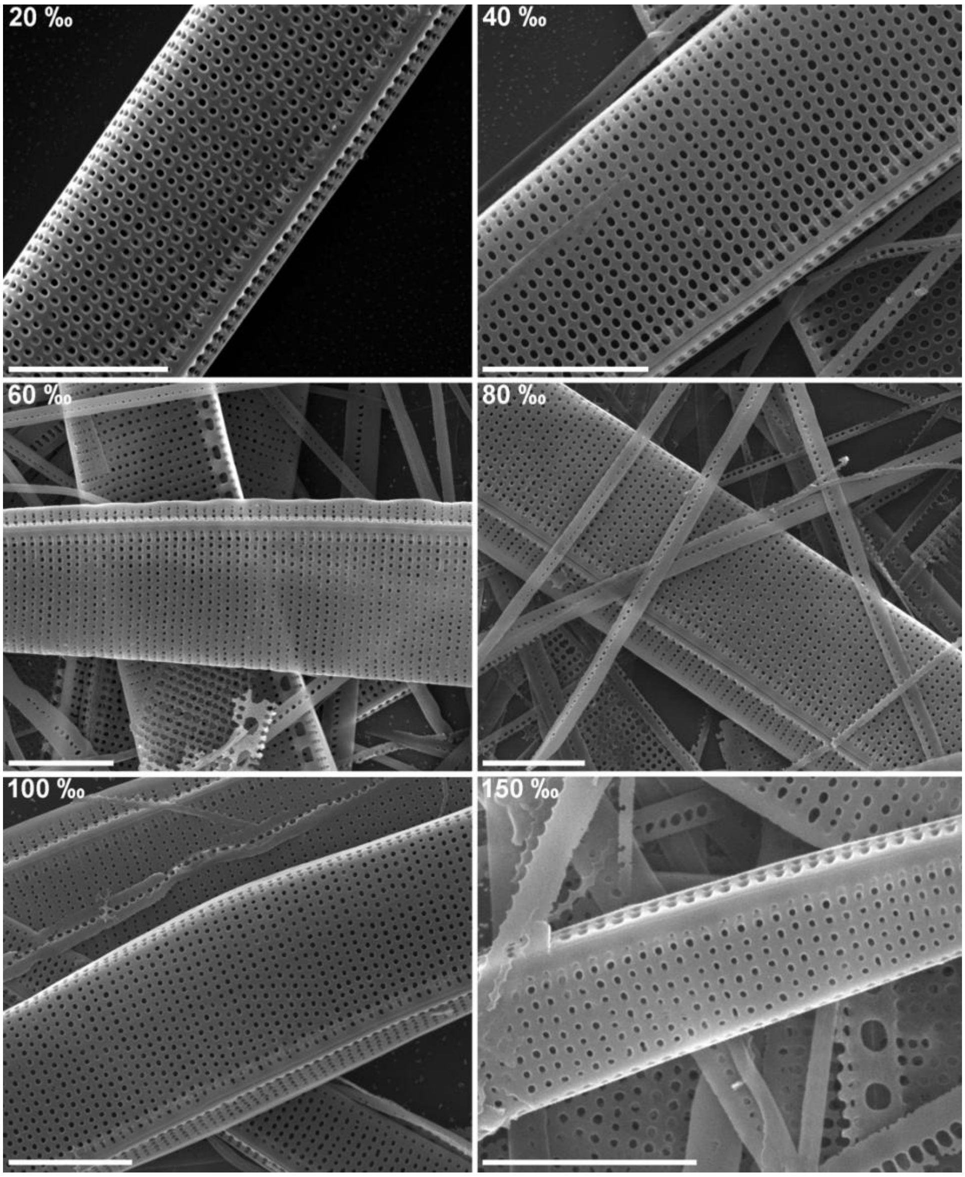

Figure S4. SEM images of cleaned *Nitzschia* sp. frustules grown at different salinities of the nutrient medium. Scale bar, 10 μm.

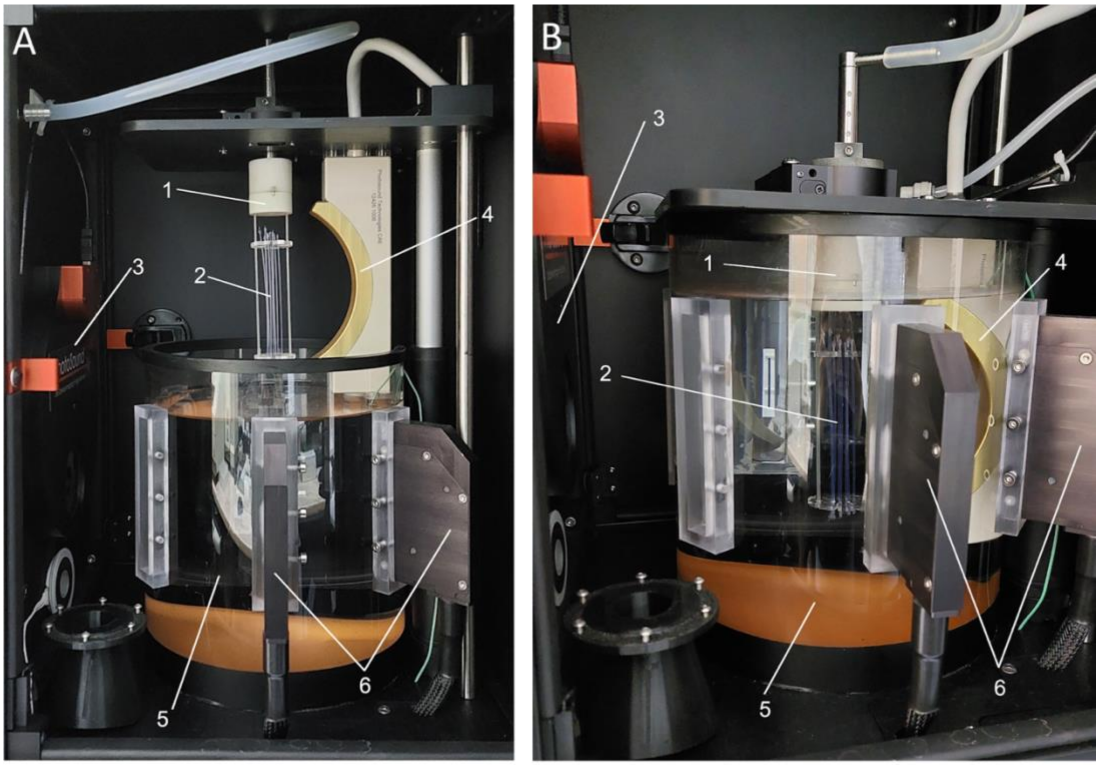

Figure S5. A - Imaging chamber of TriTom photoacoustics tomograph when the samples are out of a water tank, B – when samples are immersed in a water tank. 1. Sample holder. 2. Sample suspension in tubes. 3. Fluorescence filters' wheel. 4. Array of PA transducers. 5. Tank filled with bidistilled water. 6. Laser waveguards.

Absorbance and fluorescence spectra measurements

Absorbance and fluorescence spectra were recorded with an Infinite M Nano+ (Tecan Trading AG, Switzerland) dual-mode microplate reader. The fluorescence was excited at 460 nm, and the emission was observed in the range 620–750 nm. The absorbance spectra were collected in the wavelength range 400-750 nm.

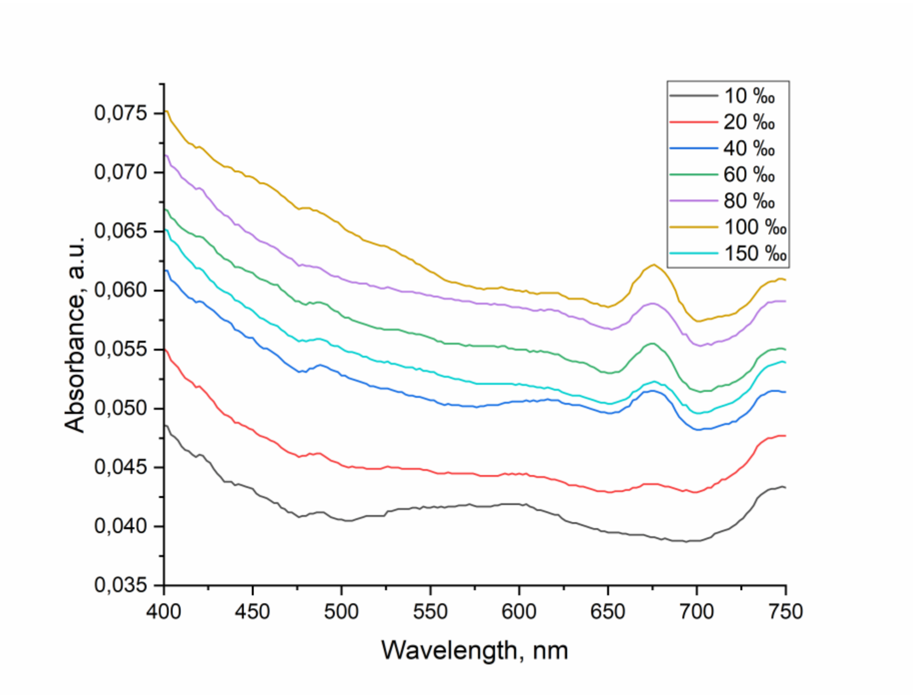

Figure S6. Absorbance spectra of diatoms in different salinity media collected at stationary phase.

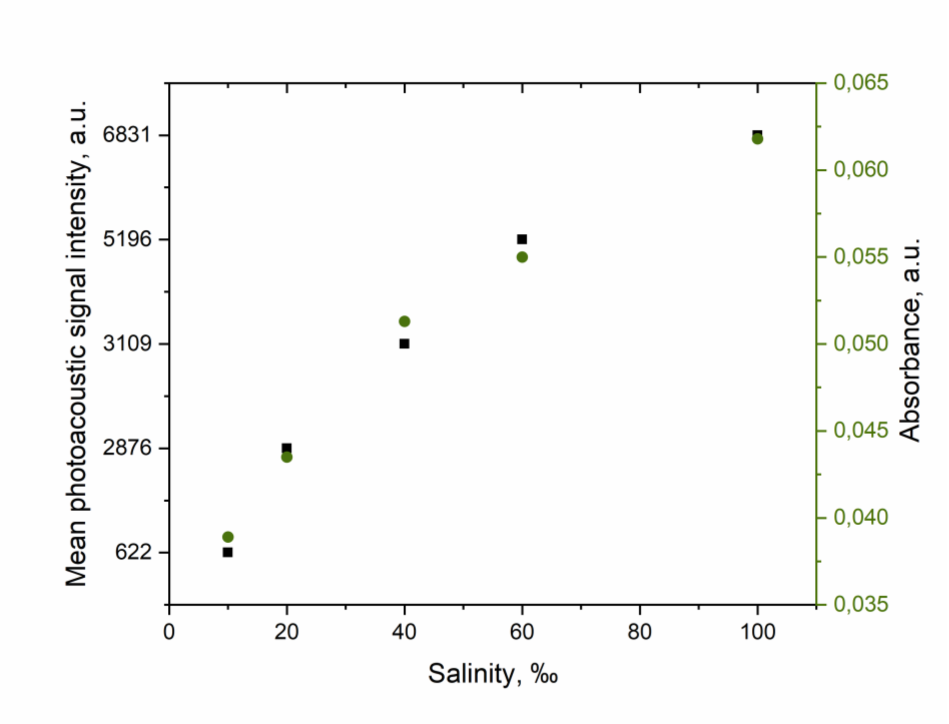

Figure S7. Comparison of absorbance of diatoms at 680 nm and mean photoacoustic signal intensity at 680 nm.

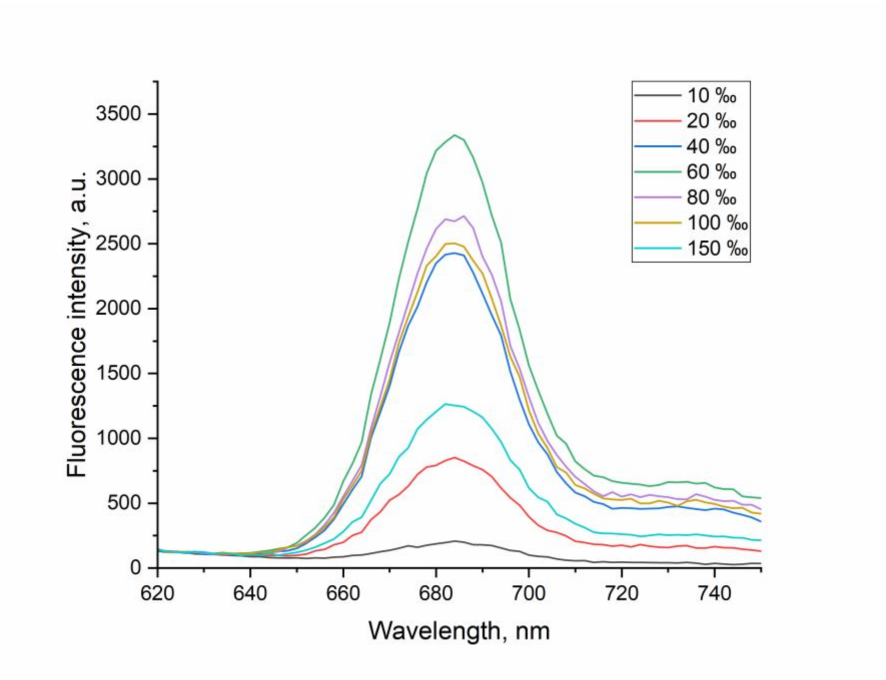

Figure S8. Fluorescence spectra of diatoms in different salinity media collected at stationary phase. Excitation wavelength: 460 nm.

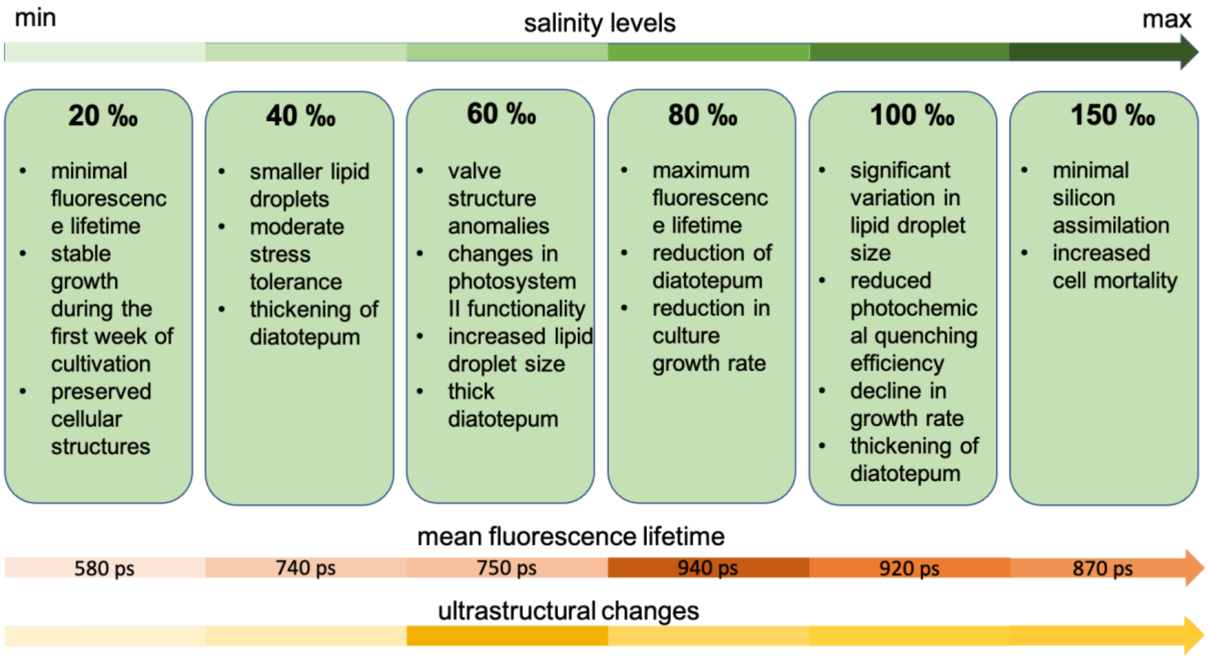

Figure S9. Schematic illustration demonstrating the salinity induced changes in ultrastructure and fluorescence dynamics in marine diatom *Nitzschia* sp.
